# Multimodal teaching analytics: the application of SCORM courseware technology integrating 360-degree panoramic VR in historical courses

**DOI:** 10.1038/s41598-023-46229-2

**Published:** 2023-11-02

**Authors:** Yanxiang Zhang, Ya Wang, Guimin Fan, Yi Song, Yunfeng Hu

**Affiliations:** 1 Department of Science and Technology Communication, University of Science and Technology of China, Hefei, 230022 Anhui China; 2 School of Marxism, University of Science and Technology of China, Hefei, 230022 Anhui China

**Keywords:** Cognitive neuroscience, Reading

## Abstract

History courses are an essential part of a national education. The application of traditional courseware's media forms in education still requires further development and refinement. Herein, we report on a history courseware mode that integrates various historical teaching media, including 360-degree VR, paintings, maps, infographics, text, audio, and videos, based on the SCORM standard. These media elements are used to provide learners with a multimodal learning experience in history courses. We monitor the learning effects using EEG and questionnaires. The results show a significant improvement in our multimodal courseware technology compared to traditional courseware.

## Introduction

History courses are an integral part of a national education but are often dull and tedious, with monotonous and outdated teaching methods. It takes work to motivate students to learn history. Thus, the reform of the history curriculum model has become a pressing matter of the moment^1,2^.

In history teaching, students often can only understand historical facts through graphic descriptions in books or traditional multimedia courseware. However, such simple graphics cannot easily arouse students’ visual perception and memory abilities under certain circumstances^3^. Because the human visual system is three-dimensional, while traditional teaching media are two-dimensional or even one-dimensional, the dimensions of cognitive experience are relatively low, which may lead to relatively unsatisfactory teaching effects^4^. History teaching involves more situational and story-telling knowledge. Using the method of historical field trips, which allows students to experience historical deeds and historical sites, can deepen historical memory and knowledge. However, there are many limitations to this method. Painted representations of historical events, immersive VR (virtual reality) representations of monuments, maps of spatial clues regarding the development of historical events, the presentation of information maps of historical facts, and the application of historical image data can provide multiple cognitive contexts to construct historical knowledge. However, previously, these methods often existed in mutually isolated media forms, and these split forms of media caused students to be distracted and disoriented.

Virtual simulation technology is becoming increasingly crucial in history education around the world^5^. However, teaching and learning methods based on virtual reality simulation still require in-depth research^6^, as the immersive history teaching method in the past has brought about both opportunities and challenges^7^. One of the advantages of virtual reality in history teaching is that by creating a realistic environment, educates can fully immerse themselves in a simulated virtual environment, and this effect of such immersive experiences can facilitate learning. Some studies suggest that a high degree of visual realism benefits people’s memory of history^8^. Virtual simulation technology provides an immersive virtual world for history education, which can inspire a better form of "learning history by experiencing history." In traditional teaching settings, history education usually includes text, photos, videos, websites, etc. At the same time, virtual reality helps students experience and generate a sense of history, which can be mapped to reading and memory in traditional history education. Such experiences help students learn history more interestingly and effectively by better "experiencing history"^9^. Therefore, combining traditional teaching with virtual reality technology makes it possible to find the best combination of the two teaching methods and give full play to their common advantages^10^.

**Figure 1:**
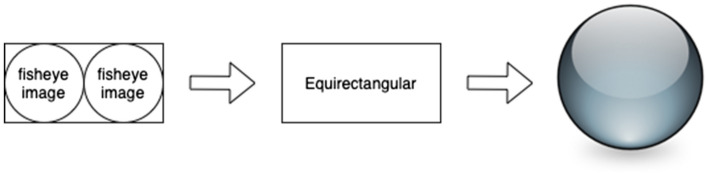
Schematic diagram of 360-degree panoramic VR.

360-degree panoramic VR are images or videos with 360 degrees of horizontal and 180 degrees of vertical field of views^11,12^; this is a way to restore and display a real scene of omnidirectional interactive viewing by splicing one or more groups of photos taken by a camera ring in a 360-degree manner into a panoramic image through computer technology (Fig. 1). Users can observe every corner of the scene by rotating their heads or body to achieve an immersive feeling^13^. 360-degree VR technology is gradually attracting the interest of researchers because it does not require complex scene construction, its production is more straightforward, it has the characteristics of VR immersion, interactivity, and imagination, and its scene display is more realistic^14^. Authentic 360-degree scene restoration based on panoramic video and virtual reality content has the characteristics of solid authenticity, high immersion, a short production cycle, low cost, and good compatibility. With the continuous integration and development of immersive technology and education, panoramic videos based on virtual reality technology have provided new vitality for educational innovation^15^. One of the facilitating effects of the learning environment provided by 360-degree VR technology on students’ knowledge and skill acquisition is the simulation of specific scenes. Common simulation scenarios mainly involve scenarios that are not or cannot be experienced in real life, such as historical and dangerous scenarios. Realistic scene simulations can provide students with an immersive experience and stimulate their learning enthusiasm^16^.

The use of online courses has been universally adopted^17^. Only by constantly innovating new teaching application forms in courseware can we better adapt to online education development, promote online education improvement, and improve online education learning efficiency. SCORM (Sharable Content Object Reference Model) is a widely used online courseware standard that supports learning management systems^17^. In China, due to the combination of SCORM courseware and learning management systems to provide the management of learning progress, SCORM courseware is widely used by MOOC platforms to produce online video courses. 360-degree panoramic VR technology has unique educational advantages, but the SCORM standard does not support such technology. Using external links to VR content outside of the courseware can cause students to be distracted. Therefore, some researchers have attempted to develop technologies to integrate 360-degree VR technology into SCORM courseware^18^. Adobe has also added support for 360-degree VR technology in its SCORM-capable courseware production tool known as Captivate 2019.

The use of 360-degree Panoramic VR videos in history teaching has gained increased attention in recent years due to its potential to enhance students’ learning experiences. While challenges remain to be addressed, such as the effective integration and management of different types of historical media, the development of SCORM courseware technology provides a promising solution. Our paper proposes an extensive development and innovative application based on the SCORM (Sharable Content Object Reference Model) standard, with a focus on implementing courseware technology that integrates VR, graphics, infographics, maps, audio, video, text subtitles, and time series composite data based on the SCORM standard and applying this technology to the online education portion of Chinese history courses, thereby making relevant knowledge easy to absorb. We combine multimodal theory to conduct a controlled experiment between the courseware based on SCORM 360-degree panoramic video integration and traditional courseware. The methods of EEG and questionnaire surveys indicate that this new courseware form brings about new learning experiences and effects to online history courses.

## Results

Through brainwave experiments, this study not only recorded the changes that occurred in learners’ attention while watching different forms of online courseware in real time but also analyzed the learning effects experienced through this format and explored the impact of traditional online courseware and SCORM courseware integrated with 360-degree panoramic VR video on learning attention and learning effects. We obtained data on the alpha brainwaves of 30 people and compared the data from two control groups. We found that students who watched the panoramic SCORM courseware had a higher mean alpha EEG value (6.1) than those who watched a MOOC video (3.4).

**Figure 2:**
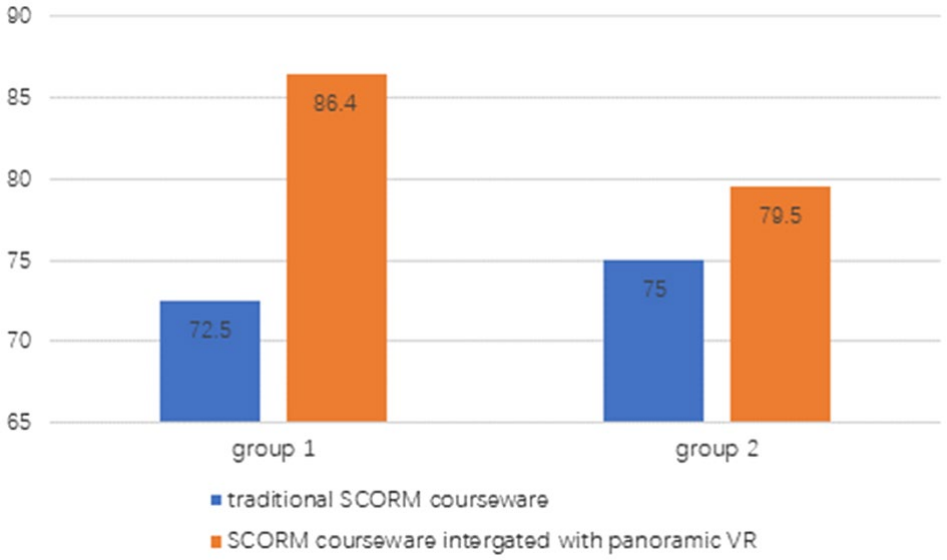
Comparison of the average scores of the four classes.

**Figure 3:**
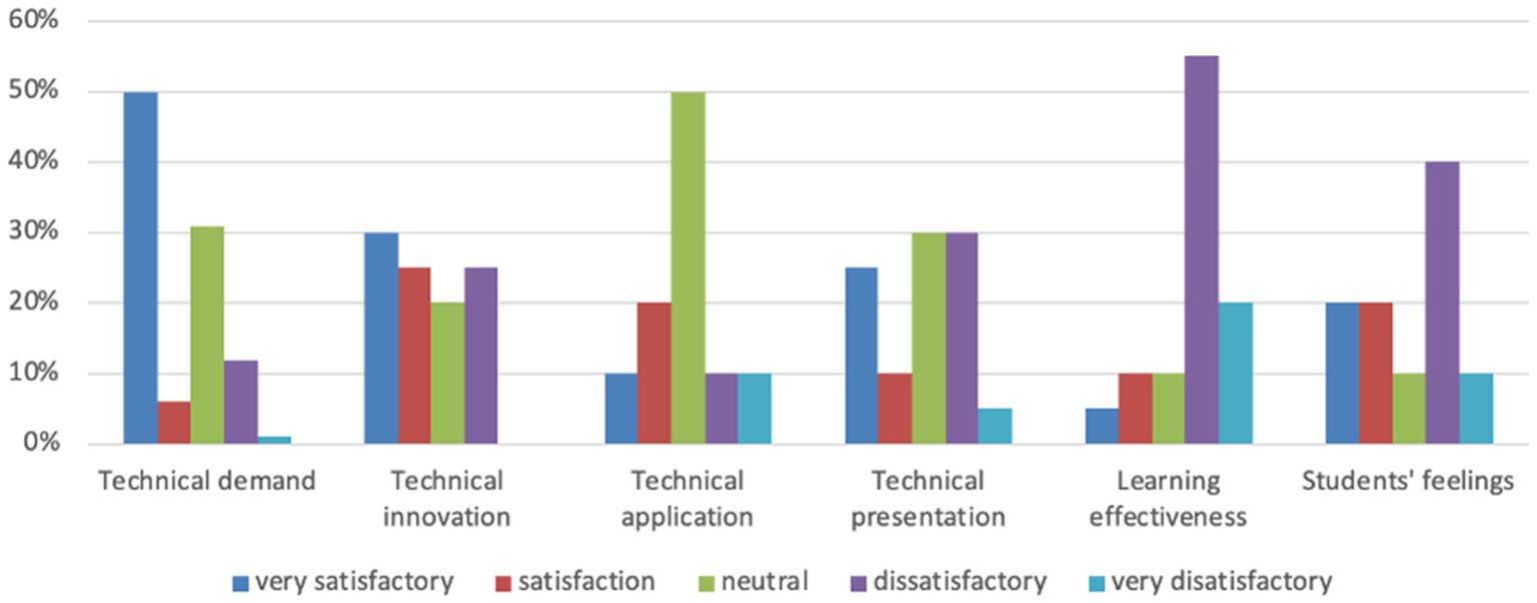
Feedback on the application of traditional courseware.

**Figure 4:**
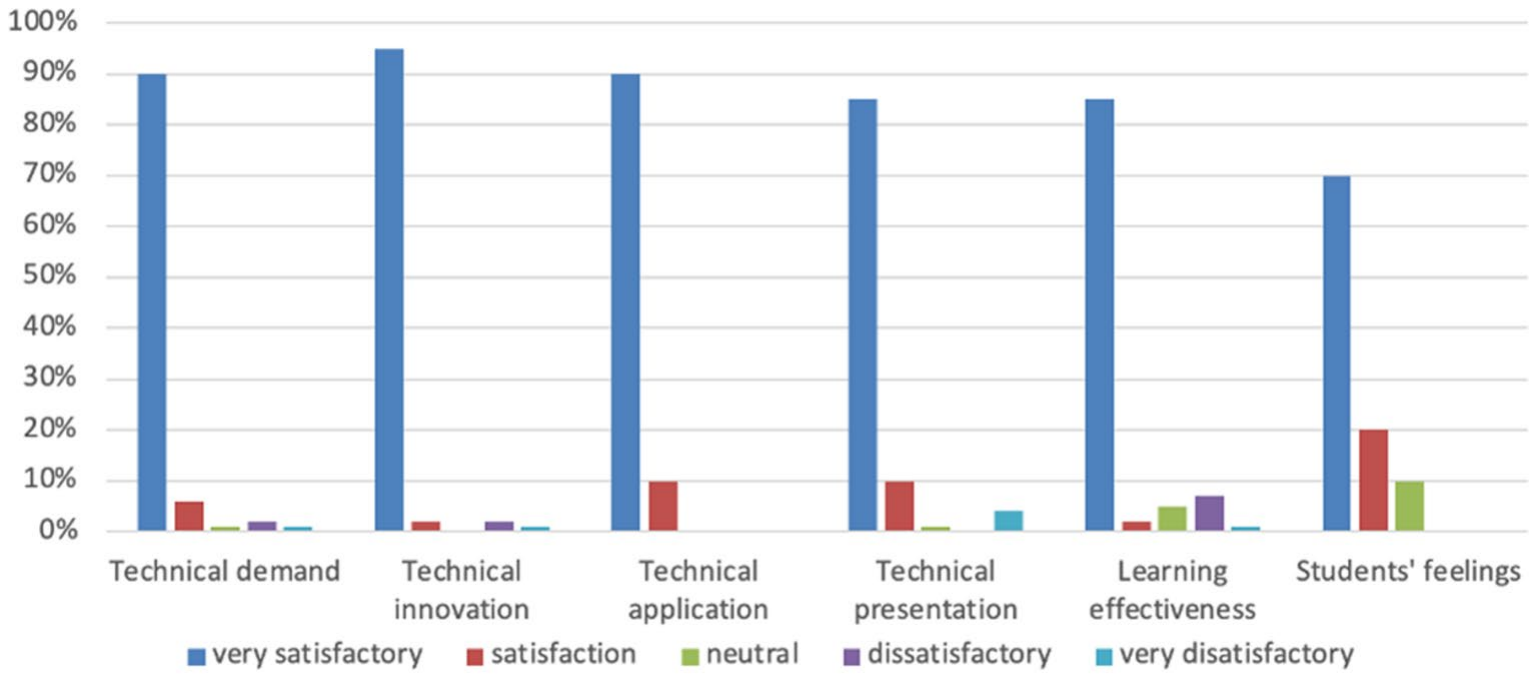
Feedback on the application of SCORM courseware for panoramic VR video.

We compared the academic performance of students using a 2 × 2 model for half of a semester (Fig. 2). We can see that the average score of students who used SCORM courseware integrated with panoramic VR was significantly higher than that of the students who used traditional courseware. Compared to traditional courseware (Fig. 3), we can see that the satisfaction level of students who used the new courseware exceeded that of most other students (Fig. 4). According to the open-ended questions, most students believed that the teaching reform of online history courses is crucial. Most students also believed that SCORM courseware integrated with VR can help them better memorize the timeline of historical events and cause them to establish a stronger historical emotional identity. The historical location information presented by the new courseware is more accurate, and the learning effects are more pronounced. Compared to traditional online teaching forms, students hoped that the content of more history courses would be presented in this courseware format. Based on the feedback, the authors also discovered advantages of VR-based SCORM courseware compared to traditional SCORM courseware, that is, newer formats, the presentation of richer visuals, richer interactions, better immersion, and a better sense of historical and emotional identity. Most students reflected that the presentation of 360-degree panoramic VR videos innovates the courseware format in online courses. This courseware model can more realistically present historical scenes and be linked to the teacher’s explanation, thereby deepening students’ memory of historical events and making the online class atmosphere more attractive. Students can participate more in learning and thinking, which will help to deepen their unique understanding of history. Due to the management of the new learning system, students’ distraction level and level of recreational behaviors have decreased. Some students stated that the 360-degree panoramic VR video format, as a relatively novel courseware model, can help to enhance their interest. The historical images provided in such videos are more attractive and cause their attention to be more focused. Most students support applying SCORM courseware technology with VR to future online learning.

We have evaluated and investigated our work from the perspective of teachers. According to teachers’ feedback, the cooperation between SCORM and LMSs has a unique constraint and management effect on students’ behavior in online courses. By setting learning times, the behavior of browsing entertainment websites or other entertainment during online courses is reduced, and students’ attention in class is greatly improved. Courseware technology can innovate the online teaching mode, which can give students an immersive history feeling in teaching. The historical scenes provided in panoramic VR videos effectively attract students’ interest in the class, making the atmosphere more relaxed and pleasant. Thus, this approach breaks from traditional history online courses’ dull and rigid teaching mode and meets the teaching objectives and requirements.

## Methods

**Figure 5:**
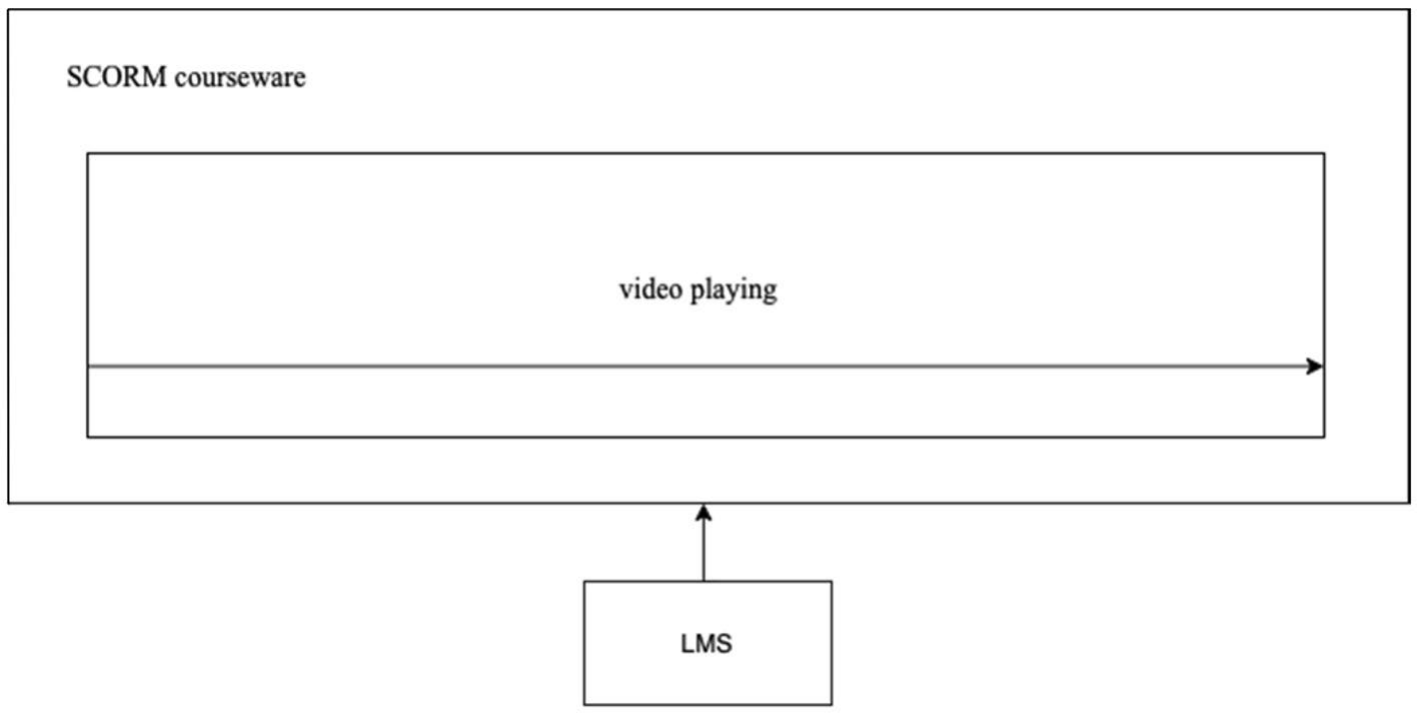
Traditional online SCORM courseware playing mode.

SCORM is a widely used online courseware standard that supports learning management systems^19^. However, traditional SCORM courseware supports only video media; it does not support VR presentations (Fig. 5). The authors propose the technical idea of integrating various media forms, such as 360-degree VR, into online courseware based on the SCORM standard. 360-degree panoramic VR, paintings, maps, infographics, audio, video, and other elements can be arranged as independent SCO units in the SCORM courseware^18^. This integration fully uses the advantages of 360-degree VR to enhance participation in online history courses and deepens the understanding of the teaching content through immersive teaching methods.

**Figure 6:**
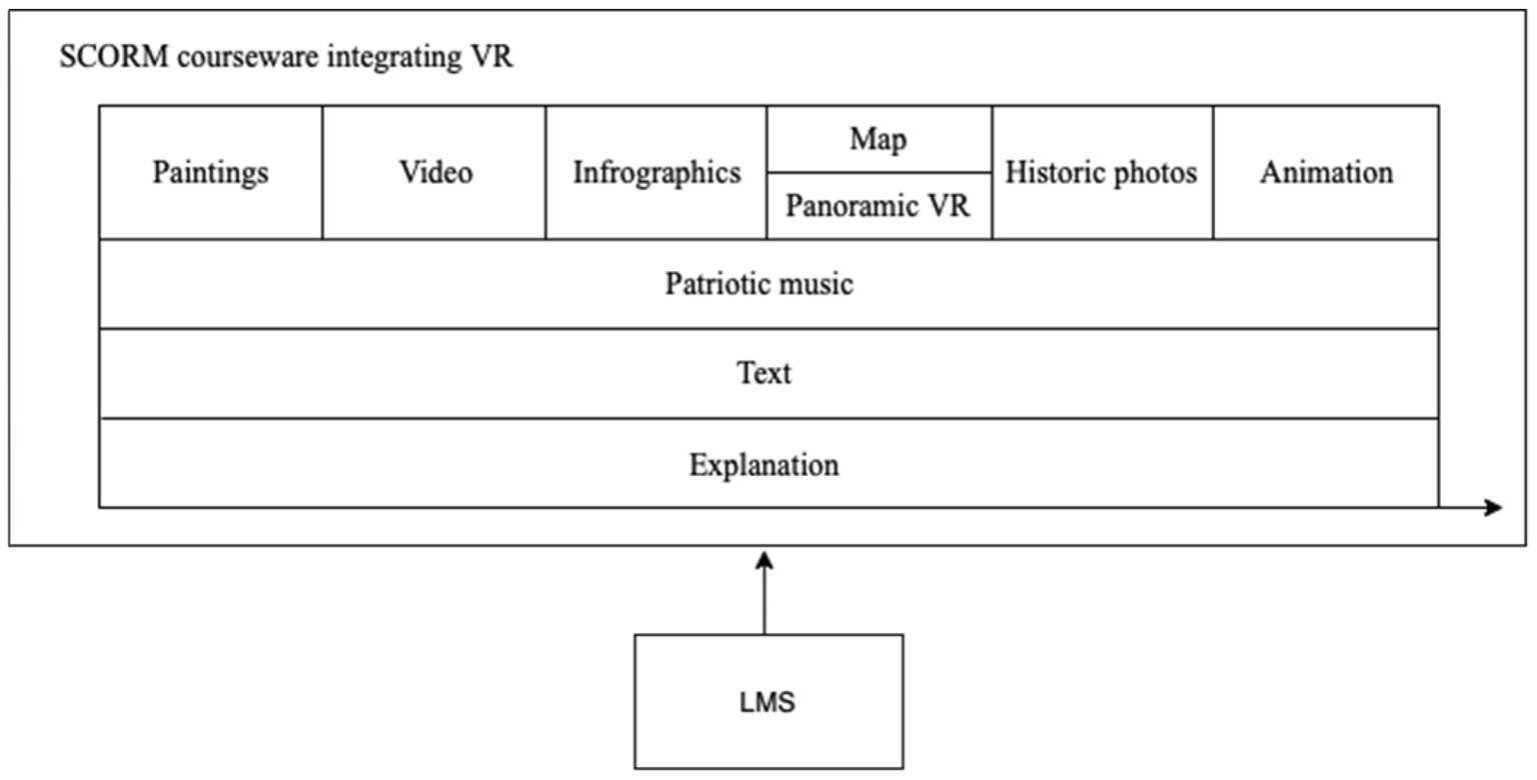
SCORM courseware integrating 360-degree VR.

Users can directly integrate and apply the internet- and VR-based controllable VR panoramic video SCO units on their browsers (Fig. 6). This mode allows learners to experience 360-degree panoramic VR without opening external links, thereby avoiding the sense of fragmentation that comes with the distractions of VR experiences. This courseware can be managed and monitored through a learning management system (LMS), making the experience of panoramic VR units completely controllable. A learning management system can manage learning progress and automatically save students’ learning records.

We will use the developed SCORM courseware technology to integrate 360-degree VR videos into the production of online historical science courseware and organically combine teachers’ teaching explanations with 360-degree VR videos. In addition to presenting spatial scenes of historical relics, 360-degree VR video also achieves the representation of historical scenes through paintings, the presentation of historical facts, and the development process of historical events through information maps. In addition, it combines the superposition of interactive characteristics such as text titles, phonetic explanations, event simulations, and clickable buttons that enable one to view more specific information in different locations of VR images; these aspects help students achieve multidimensional graphical interpretations and information transfer based on 360-degree VR video and realize a rich media display of historical content. Specifically, maps can help students understand macro clues related to the occurrence and development of historical events. Historical paintings can help immerse students in on-site simulations and representations of historical events. At the same time, such paintings can complement the VR experience of historical sites to bring students an immersive experience of the actual space of the scene where historical events occurred and make students feel the multimodal cognitive experience brought about by historical facts. The animation simulation presented on a VR screen can help students reproduce the behaviors and actions of historical figures in real physical space so that students can better feel the atmosphere around and understand historical events. Audio explanations can explain the basic historical information of an event and give students a deeper understanding of history. Subtitles can serve as the direct and high-intensity presentation of important historical information. Background music mainly refers to patriotic music related to history. By playing background music that matches historical events, students can be more fully immersed in the context of historical events.

**Figure 7:**
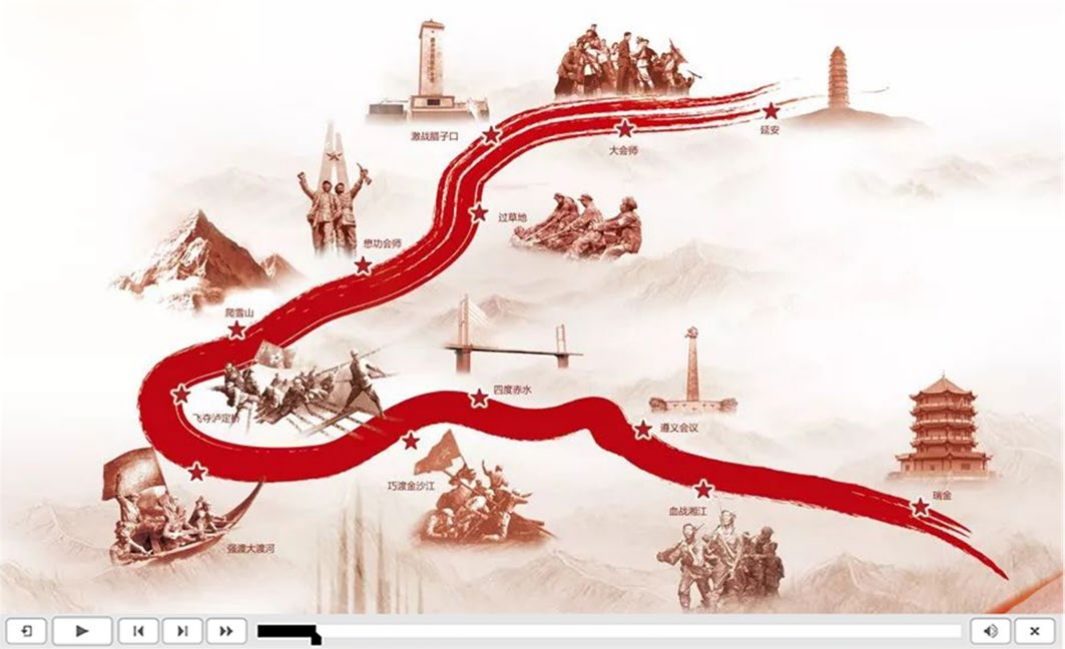
The infographic of historical sites available for VR.

First, as shown in Fig. 7, the infographic gives the audience an abstract and quick interpretation of historical facts and a macro impression of a concept.

**Figure 8:**
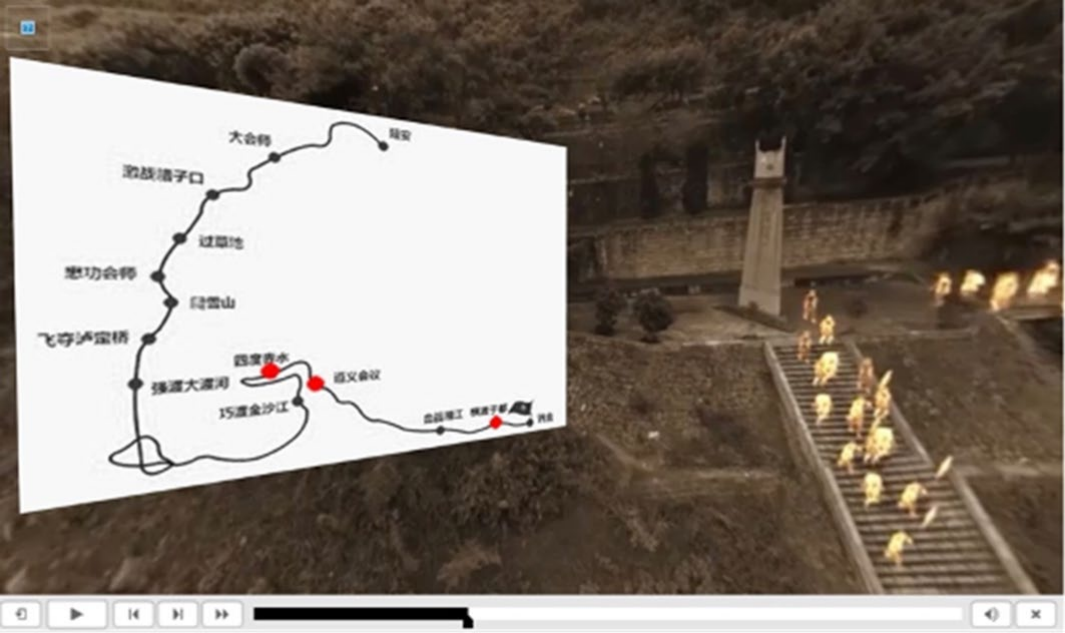
Panoramic VR image of the Long March.

Second, as shown in Fig. 8, a road map is superimposed on a 360-degree VR picture of a historical heritage scene. The course offers historical VR maps that accurately capture time and space to help users explore history in a vivid and immersive atmosphere. The progress of historical events is prompted by using the road map based on the current scene. When students watch this section, they will first see this photo. Clicking on any interface can pause the panoramic video, facilitating students to develop not only an impression on the geographical location of historical events but also a more specific understanding. 360-degree panoramic VR is an immersive experience. In traditional online courseware, the experiences related to and the teaching of panoramic VR are separated, which often requires separate 360-degree panoramic VR experiences and interactions, leading to student distraction. However, the duration of each segment of panoramic VR integrated into SCORM has been set, thus avoiding the above issues.

**Figure 9:**
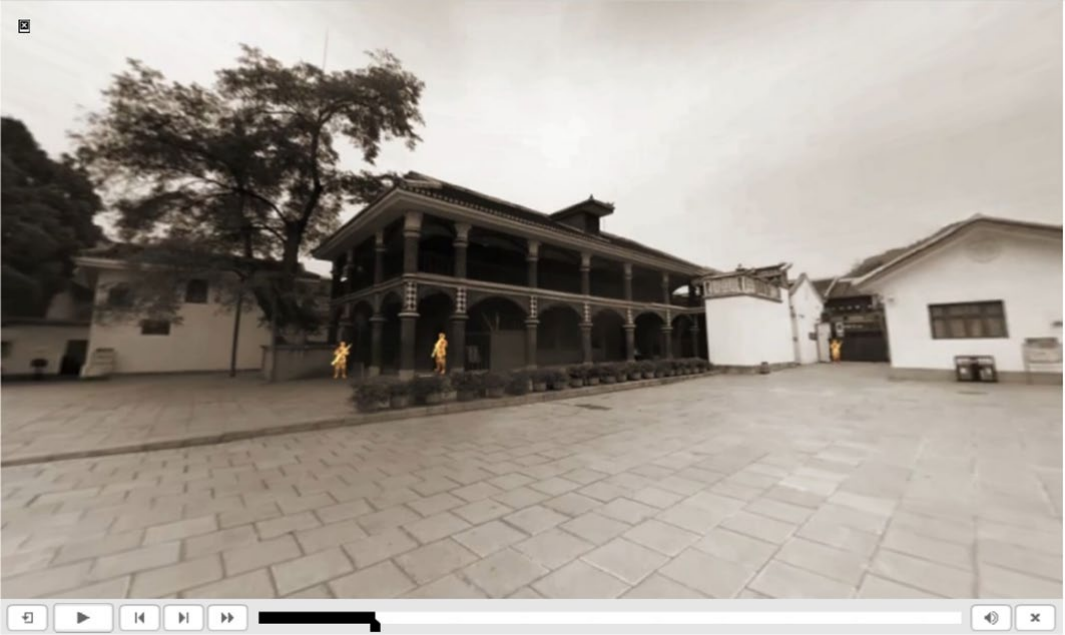
Animation of an historical scene superimposed on the panoramic video.

Third, as shown in Fig. 9, through historical VR animation, users can witness and simulate historical events in actual scenes of historical landmarks. The courseware also shows videographic interpretation units in the courseware. There is a playback progress bar positioned below the scene and a playback control interface, which can display the current playback progress or allow students to fast-forward to the next section or return to the previous section. These sections may be presented as a video or panoramic VR event. The provided infographics can help students interpret historical facts both abstractly and quickly and observe the learning content more directly.

**Figure 10:**
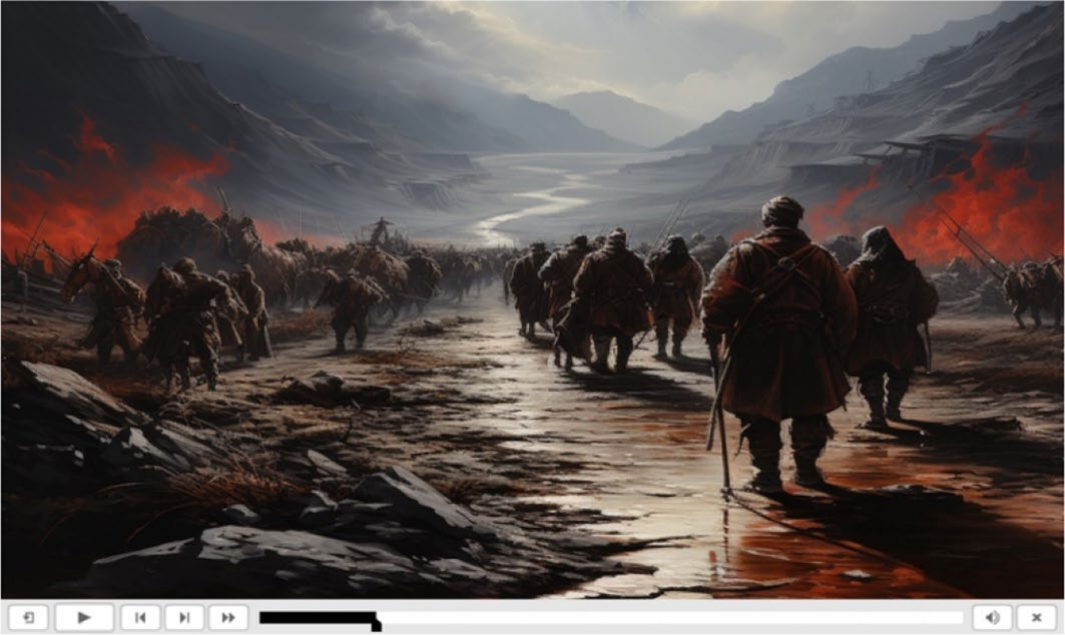
Historic paintings help present realistic and replayed scenes.

Likewise, another aspect of the issue is that historical paintings help fill in the gaps of events that lack visual recording, presenting realistic and replayed scenes. These features provide a unique and comprehensive experience that can supplement research papers with an accurate and detailed historical narrative (Fig. 10).

Finally, integrating 360-degree panoramic VR technology into web-based courseware will bring a new model into the development of online history teaching. 360-degree Panoramic VR requires less capacity and is easy to transmit on the internet, representing the future development trend of online courseware. Our work enables 360-degree panoramic VR video to be continuously integrated into SCORM courseware, while separate virtual reality experiences can result in the separation of students from the teaching process. The new model combines the theory of online history teaching with scenes that help students experience, e.g., the Red Revolution in a virtual reality format, which is beneficial for helping them memorize and learn historical knowledge. The application of SCORM courseware integrating 360-degree panoramic VR videos in online teaching enables students to participate in the teacher’s explanation and experience VR scenes during the explanation process. At the same time, aspects of such VR experiences can be set within the SCORM learning system management mode, such as experience duration, nodes, and content, which can help better manage students’ learning behavior in the teaching process.

In this experiment, undergraduate students from two classes were recruited as subjects and randomly divided into two experimental groups, with 15 participants in each group and an average age of approximately 20 years. Experimental research methods were adopted, and the tools used mainly included an EEG instrument, physiological saline felt sensor, normal saline, USB receiver, computer, etc. The EEG device used in this experiment was an Emotiv EPOC X wireless portable brain wave instrument, which collects EEG waves to generate EEG signals and then converts these EEG signals into data that can be analyzed to measure performance metrics.

**Figure 11:**
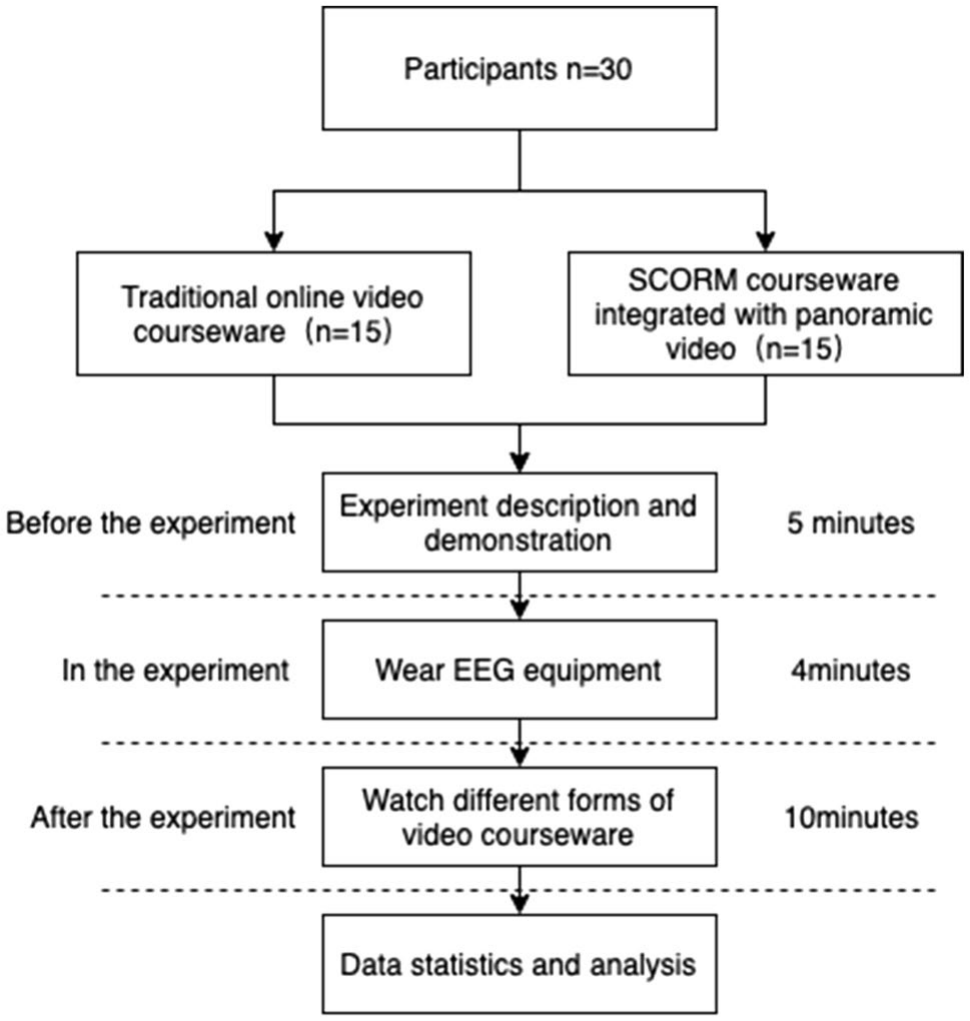
The experimental process.

Before the experiment, participants were randomly assigned to two different groups. To ensure the smooth progress of the experiment, the researchers first explained the entire experimental process and precautions to the participants. They demonstrated the wearing of the brain wave instrument and told participants to stay relaxed during the experiment. At the beginning of the experiment, the subjects wore the EEG instrument to collect brainwave data after watching different video courseware. All subjects were willing to participate in the experiment and had the right to leave. We pseudonymized the data and kept it confidential (Fig. 11).

This experiment adopted a single-factor interactive test experimental design. The independent variables were two forms of online courseware, namely, the online courseware provided in a Chinese MOOC and the VR SCORM courseware, which offered historical scenes of the Long March of the Red Army. The dependent variable consisted of students’ attention and learning effects. The experiment was conducted in a quiet and spacious laboratory on the north campus of the University of Science and Technology of China. The researchers observed the brain wave data presented in the computer in real time to discover and handle problems in the experiment.

**Figure 12:**
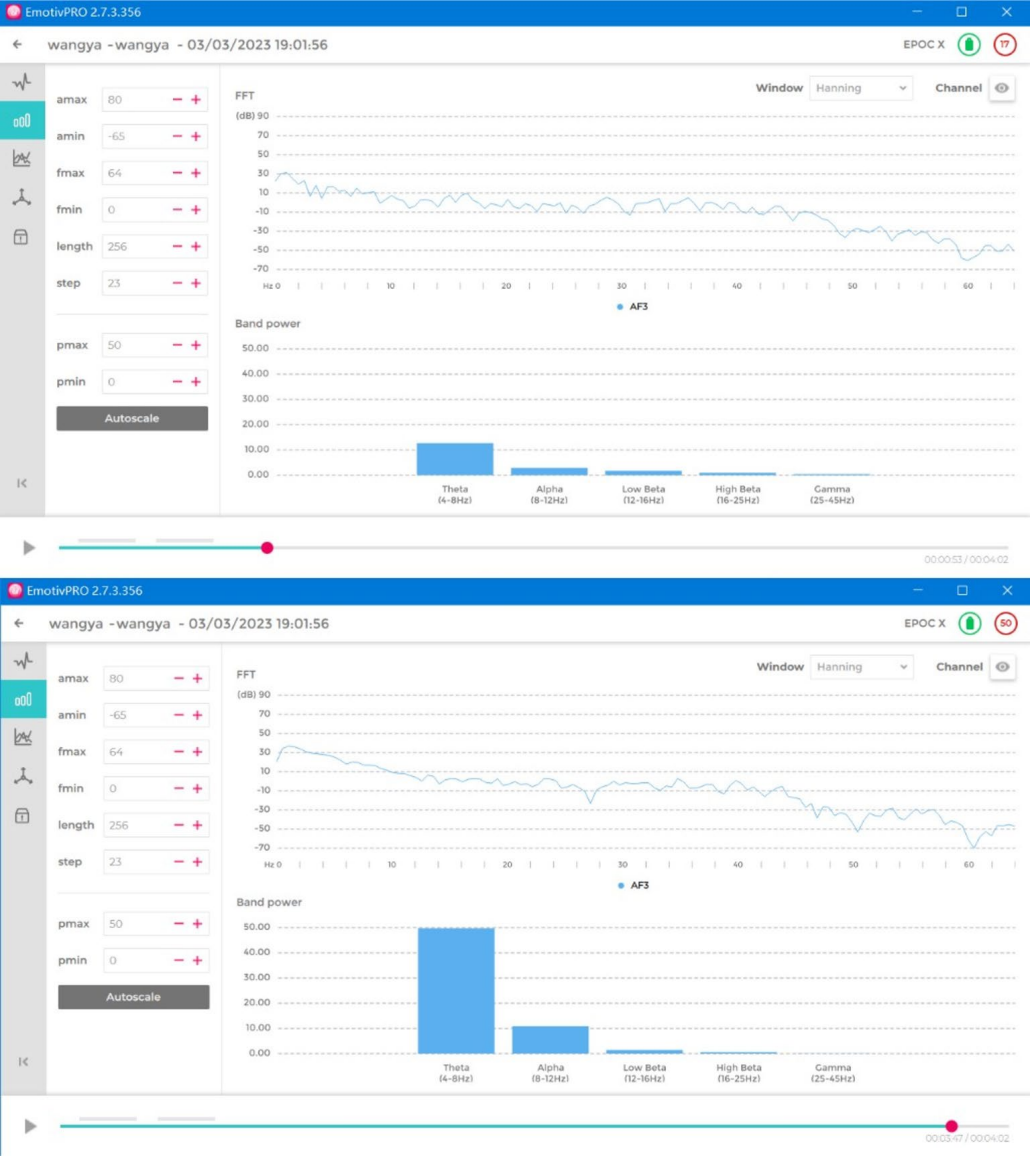
Average EEG values of students watching the panoramic video SCORM courseware (6.1) and the MOOC videos (3.4).

EEG data are divided into four waves: alpha, theta, beta, and gamma. Alpha brain waves are those generated when the human body is in a naturally relaxed state or the brain is in an active state. Alpha brain waves have a particular impact on learning. Only when the human brain emits alpha brain waves can it generate an excited state that allows it to think and memorize knowledge. Keeping the alpha brainwaves in an active state is more conducive to learning. EEG data (Fig. 12) were analyzed during the tracking task, and the relevant alpha frequency fluctuation and change characteristics were analyzed. We observed the data output from the EEGs on the computer and observed and visualized the average values.

We sampled 140 undergraduate students from four classes in the computer science department of a university. The course content consisted of the historical event of the Long March of the Red Army, and the learning time consisted of 2 h. They were approximately 35 students in each of the 4 classes. We compared the students' academic performance using a 2 × 2 model throughout half of a semester.

Curriculum evaluation is crucial in the teaching system and consists of detecting, evaluating, and judging the value of teaching processes and results. At the same time, teaching evaluation feedback positively impacts teachers and students. Therefore, to understand the teaching effect of SCORM courseware integrated with 360-degree panoramic VR video in history courses, we designed experiments and questionnaires that aimed to investigate students’ feedback after watching SCORM courseware with 360-degree panoramic VR video, and we used diverse evaluation methods to evaluate the application of the new model. The new courseware technology was applied to the history courses taken by computer science and technology undergraduate students from the class of 2022 during the study period. We designed 18 questions from six aspects: technological needs, technological innovation, technological application, technological demonstration, learning effects, and students’ feelings.

## Discussion

Multimodal learning uses multiple sensory channels, such as visual, auditory, and kinesthetic channels, to process and retain information. Providing learners with multiple learning modes can enhance their comprehension, retention, and application of knowledge. Each unique attribute becomes a "modality," and learning using integrated multimodal information is multimodal learning. Different modalities can exist in the same media^20^.

Several studies have systematically summarized the development process, application scenarios, and technical methods of multimodal education, providing rich reference information for researchers in related fields. Callaghan et al. introduced how to use multimodal analysis technology in education, focusing mainly on the fusion of various data sources and the correlation between multimodal analysis technology and learning analysis. The article also introduced the development and application of multimodal education, covering various teaching methods and learning materials while emphasizing integrating multimodal learning technology into teaching. According to scientific research, multimodal learning can help students gain a more comprehensive understanding of historical events and figures and stimulate their interest in learning history. Previous studies have indicated that multimodal learning methods can significantly enhance students’ interest and participation in learning history and promote knowledge retention and understanding. Multimodal resources, including images, videos, and audio, can also help to facilitate students’ comprehension and memory of historical events while improving their critical thinking and judgment skills. Therefore, it is evident that multimodal learning approaches are vital in enhancing history learning outcomes. The use of multimodal resources for teaching has become a hot topic in education and is gradually being implemented in various fields^20–23^.

In SCORM courseware, 360-degree panoramic VR, paintings, maps, infographics, audio, and video can be presented in multiple ways, such as different approaches to timing and juxtaposition, to help bring users a multimodal cognitive learning experience that includes the immersive experience of virtual reality, the reproduction of historical images without image records through paintings, the display of spatial clues suggesting the development of historical events through geographical maps, the analysis of historical facts through infographics, the insertion of historical audio and video into historical scenes, and the presentation of subtitle and narration information in the text. The data from various modes help mobilize multiple sensory perception systems and complement each other, thereby building up students’ cognition of historical knowledge through multiple cognitive dimensions and modes and enhancing students’ complete understanding and processing of historical knowledge.

According to the theory of multimedia learning, the brain processes information through two systems, i.e., one for verbal materials and the other for visual materials. By presenting words and pictures together, multimedia learning takes advantage of the brain's dual-channel processing and maximizes the use of information processing resources^24^. This type of multimedia learning better facilitates learning tasks for individuals. By integrating panoramic virtual reality (VR) visual materials with ample verbal materials, SCORM courseware technology has emerged as a practical approach for applying ideological and political courses. It provides learners with an optimal learning path, making it an ideal mode for enhancing the learning experience.

Based on the SCORM standard, the authors integrated history teaching media such as 360-degree panoramic videos, paintings, maps, infographics, text, audio, and video to provide learners with a multimodal learning experience. The use of EEGs and questionnaire surveys showed that applying the new SCORM courseware technology, which integrates multimodal data such as panoramic VR, in history teaching has a better learning effect than traditional courseware. This courseware technology can also be managed and monitored through a learning management system, allowing for a fully controllable experience of VR panoramic units and reducing online historical entertainment behavior. This approach solves many problems related to traditional online history courses and provides an optimized application model for online history teaching.

For this experiment, we recruited 30 undergraduate students with an average age of 20 years from two different classes, with participants being randomly assigned to one of two groups of 15 students each. The study used experimental research methods, and the tools used primarily included an Emotiv EPOC X wireless portable EEG recorder, physiological saline felt sensors, physiological saline USB receiver, and a computer. The Emotiv EPOC X can collect EEG signals and convert them into data that can be analyzed to measure performance metrics. Before the experiment, the experimenters introduced the entire experimental process and precautions to the participants, demonstrated the wearing of the EEG recorder device, and instructed the participants to stay relaxed during the experiment. The participants watched different video courseware during the experiment, and their alpha-EEG data were collected and analyzed. Our analysis revealed that the mean alpha-EEG value (6.1) of the students who watched the panoramic video SCORM courseware was higher than that of the students who watched the MOOC videos (3.4) (Fig. 12).

We conducted an experiment and survey to evaluate the effectiveness of incorporating 360-degree panoramic VR videos into SCORM courseware in history classes. A total of 140 undergraduate students majoring in computer science and technology from the class of 2022 participated in the experiment and survey. We designed 18 questions based on six aspects: technology requirements, innovation, application, demonstration, learning outcomes, and student feedback. The results showed that students were highly satisfied with the 360-degree panoramic VR video-integrated SCORM courseware compared to traditional courseware (refer to Figs. 2, 3, 4). Specifically, the first set of experimental data showed that 96.4% of the students thought that the new courseware teaching mode was "very effective" or "very effective". In comparison, the proportion of students who thought that the traditional courseware was "very effective" or "very effective" was 72.5%. The second set of experimental data showed that 79.5% of students thought that the new courseware teaching mode was "very effective" or "very effective" compared to 75% for the traditional courseware. These findings demonstrate the potential of 360-degree panoramic VR videos integrated with SCORM courseware to enhance students’ perceptual experiences and improve outcomes in history classes.

## Data Availability

The datasets used and/or analyzed during the current study are available from the corresponding author upon reasonable request. We acquired permission to utilize the images presented within our internally developed courseware during the course of this research (Figs. 7, 8, 9, 10). In addition, it should be noted that we used AIGC to create an image of an historical painting (Fig. 10). Our usage of these materials aligns with the original intent of creating and disseminating these works for educational and research purposes. Thus, there is no copyright issue present in this paper.
